# Efficacy of Sodium-Glucose Cotransporter 2 Inhibitors in Heart Failure with a Preserved Ejection Fraction: A Meta-Analysis of Randomized Controlled Trials

**DOI:** 10.31083/j.rcm2311374

**Published:** 2022-10-31

**Authors:** Yake Lou, Qi Yang, Weicong Zhang, Ying Yu, Jing Huang

**Affiliations:** ^1^Department of Cardiology, The Second Affiliated Hospital of Chongqing Medical University, 400010 Chongqing, China; ^2^Department of Cardiology, The First Affiliated Hospital of Chongqing Medical University, 400016 Chongqing, China; ^3^Department of Ultrasound, Beijing Friendship Hospital, Capital Medical University, 100069 Beijing, China; ^4^Department of Neurology, Beijing Tiantan Hospital, Capital Medical University, 100070 Beijing, China

**Keywords:** sodium-glucose cotransporter 2 inhibitors, canagliflozin, dapagliflozin, empagliflozin, sotagliflozin, heart failure, preserved ejection fraction, reduced ejection fraction

## Abstract

**Background:**

Heart failure is prevalent worldwide. Sodium-glucose cotransporter 2 inhibitors (SGLT2i) are effective in heart failure patients with reduced ejection fraction, whether SGLT2i are effective in heart failure with preserved ejection fraction (HFpEF) remains to be determined.

**Methods:**

All relevant citations in the PubMed, Embase and Cochrane databases were identified from inception to September, 2022. The primary outcome was a composite endpoint of cardiovascular death and hospitalization for heart failure (HHF). A subgroup analysis was performed according to diabetes mellitus status and the ejection fraction. Secondary endpoints were cardiovascular death, hospitalization for heart failure and all cause death.

**Results:**

Seven studies involving 11,604 patients were included in the meta-analysis. Compared with placebo, sodium-glucose cotransporter 2 inhibitors reduced the incidence of the primary outcome by 24%, with an odds ratio (OR) and 95% confidence interval (CI) 0.76 [0.69, 0.84]. For secondary outcomes, sodium-glucose cotransporter 2 inhibitors were associated with a lower incidence of hospitalization for heart failure, but not cardiovascular or all-cause death; the OR and 95% CI were 0.73 [0.66, 0.82], 0.92 [0.81, 1.04], 0.96 [0.88, 1.05], respectively.

**Conclusions:**

This study proves the clinical efficacy of SGLT2i for treatment of HFpEF patients with or without diabetes, which was mainly driven by prevention of HHF rather than cardiovascular or all-cause death.

## 1. Background

Heart failure (HF) is prevalent worldwide and causes great economic burden for individual patients and society. It can be divided into HF with preserved ejection fraction (EF) (HFpEF), HF with reduced EF (HFrEF) and HF with mildly reduced EF (HFmrEF) [1, 2]. Currently, several drugs have been demonstrated to be effective in HFrEF, however, their efficacy in HFpEF is uncertain [3, 4, 5].

Sodium-glucose cotransporter 2 inhibitors (SGLT2i) is a novel hypoglycemic agent, which have been shown to reduce cardiovascular death in patients with diabetes mellitus (DM) [6, 7]. Recent large randomized controlled trials (RCTs) have confirmed that SGLT2i can improve the prognosis of HFrEF patients [8, 9]. In view of these findings, the 2021 European Society of Cardiology (ESC) Heart Failure Guidelines recommended SGLT2i (mainly dapagliflozin and empagliflozin) as the first-line drugs for HFrEF (Class: I, Level: A) [2, 4, 8]. However, whether SGLT2i are effective in HFpEF remains to be determined. In the EMPEROR-Preserved study, empagliflozin reduced the risk of a composite endpoint of cardiovascular death or hospitalization for heart failure with EF >40% by 21%, but the efficacy of empagliflozin on HFpEF was mainly driven by patients who had an EF of 40–50% [10]. The hazard ratio of empagliflozin versus placebo in EF of 40–50%, 50–60%, ≥60% was 0.71 (0.57–0.88), 0.80 (0.64–0.99), and 0.87 (0.69–1.10), respectively. Cosentino *et al*. [11] performed a subgroup analysis and found that ertugliflozin seemed to reduce the first hospitalization for heart failure (hazard ratio (HR) and 95% CI 0.86, 0.58–1.29), but this was not statistically significant. In DELIVER study, dapagliflozin reduced the combined risk of worsening heart failure or cardiovascular death among patients with heart failure and a mildly reduced or preserved ejection fraction [12]. Therefore, we performed this meta-analysis to investigate the effects of SGLT2i on HFpEF.

## 2. Methods

Our meta-analysis was conducted in accordance with the Preferred Reporting Items for Systematic Reviews and Meta-Analyses (PRISMA) guidelines [13]. All data were collected from published papers and no ethical approval was needed. The meta-analysis was registered in the PROSPERO database (NO: CRD42021276228) [14].

### 2.1 Search Strategy

The keywords “Sodium-Glucose Transporter 2 Inhibitors”, “Dapagliflozin”, “Canagliflozin”, “Empagliflozin”, “Ipragliflozin”, “Sergliflozin”, “Remogliflozin”, “Tofogliflozin”, “Luseogliflozin”, “Sotagliflozin”, “Ertugliflozin”, “Velagliflozin”, “Licogliflozin”, “Mizagliflozin” and “Heart failure” were used to search the electronic databases of PubMed, Embase, and Cochrane Central Register of Controlled Trials (CENTRAL) from inception until September, 2022. Only citations published in English was searched (details of search process are listed in the **Supplementary Materials**).

### 2.2 Inclusion and Exclusion Criteria

The inclusion criteria were (1) RCTs, (2) the intervention group was SGLT2i and the control group was other treatment but not SGLT2i, (3) outcomes of interest were reported, (4) patients with HFpEF. Exclusion criteria were (1) animal experiments, (2) observational studies, (3) real-world studies, (4) no outcome of interest reported, (5) conference reports, (6) reviews, (7) case reports or summaries, (8) studies published in a language other than English, (9) head-to-head studies that compared SGLT2 inhibitors with other glucose-lowering agents.

### 2.3 Data Extraction and Quality Assessment

Two authors (Lou and Yang) examined the titles and abstracts to find potentially eligible studies according to the inclusion and exclusion criteria. Disagreements among authors were resolved by another author (Huang). After screening, the full text was browsed for all the potentially eligible citations. Subsequently two authors (Zhang and Yu), evaluated the risk of bias for each included study according to the Cochrane Handbook for Systematic Reviews of Interventions (version 5.1.0) [15]. Baseline characteristics and outcomes were extracted by Lou and Yang.

### 2.4 Outcomes

The primary endpoint of the meta-analysis was a composite endpoint of cardiovascular death and hospitalization for heart failure (HHF). A prespecified subgroup analysis was carried out according to the diabetes mellitus (DM) status and the ejection fraction (EF) value. Secondary endpoints were cardiovascular death, hospitalization for heart failure (HHF) and all cause death.

### 2.5 Statistical Analysis

All statistical analyses were performed using the software Review Manager (RevMan) version 5.3 (The Cochrane Collaboration, Copenhagen, Denmark) and Stata 15.1 (StataCorp, College Station, TX, USA).

The odds ratios (ORs) and 95% confidence intervals (CIs) were calculated by Mantel-Haenszel analysis to investigate the effects of SGLT2i on HFpEF.

The $I^{2}$ statistic and χ^2^ test were used to evaluate heterogeneity across trials; $I^{2}$ > 50% was considered to indicate substantial heterogeneity. The Mantel-Haenszel fixed-effects model was used where $I^{2}$ < 50%; otherwise, we would analyze the potential heterogeneity and try to eliminate heterogeneity. The Mantel-Haenszel random-effects model was used if the heterogeneity was still over 50% after adjustments. We performed sensitivity analyses to evaluate the stability and reliability of the results. A visual funnel plot was used to evaluate publication bias.

## 3. Results

### 3.1 Study Selection

**Fig. 1.**
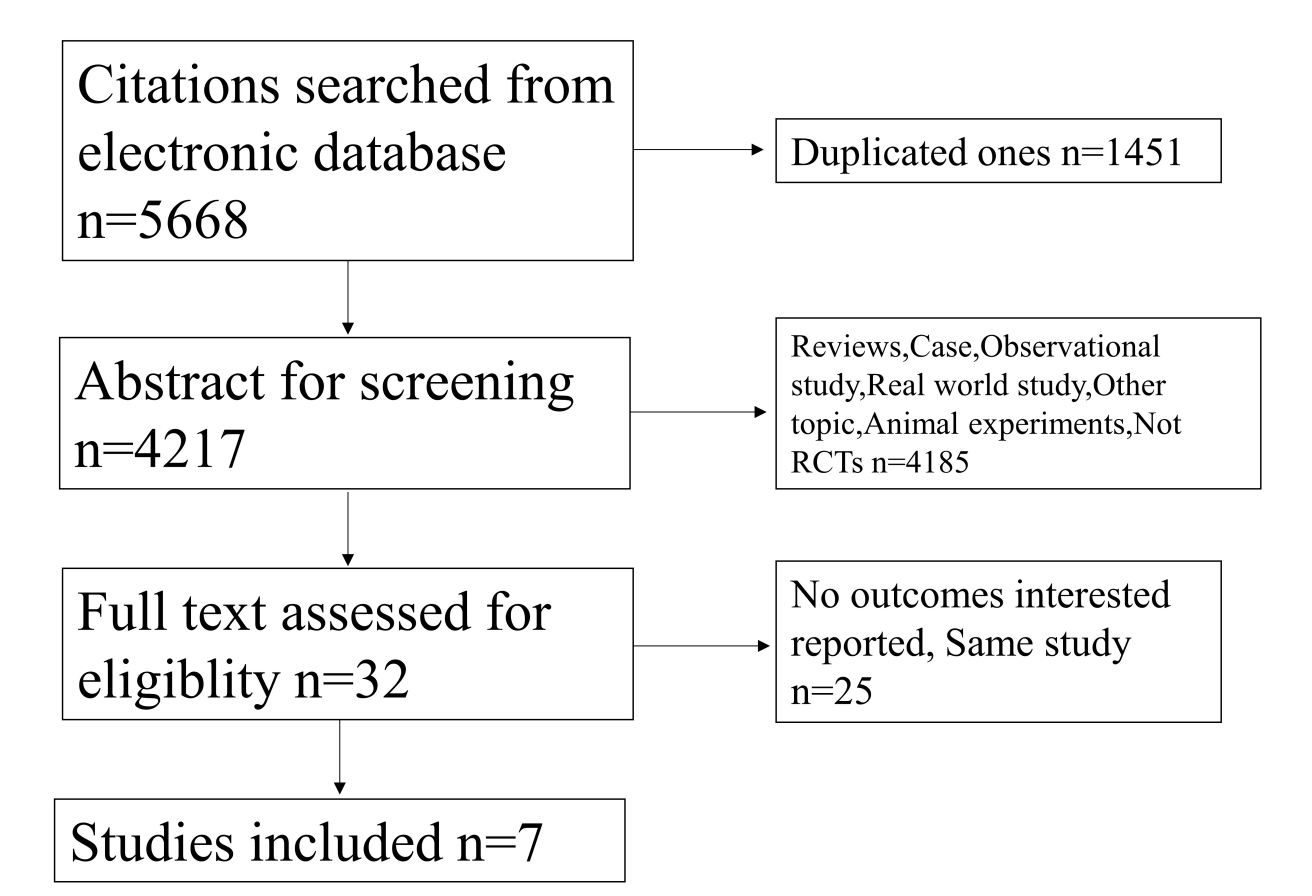
**Flowchart**.

A total of 5668 citations were identified from varied sources including PubMed, Embase, Cochrane and manual searches. After deleting 1451 duplicates, we screened potentially eligible citations by browsing titles and abstracts. Reviews, case reports, observational studies, real world studies, other topics, animal experiments and non-RCTs involving 4185 citations were also excluded. We excluded 32 other citations by full-text assessment and finally, 7 studies were included in the meta-analysis (Fig. 1).

### 3.2 Characteristics of Eligible Studies

The seven RCTs in the final analysis included 11,604 patients, with a sample size ranging from 208 to 4147 [10, 11, 12, 16, 17, 18, 19]. The shortest and the longest follow-up period was 9 months and 4.2 years, respectively. There were 6002 (51.7%) patients in the SGLT2i group and 5602 (48.3%) in the control group. The SGLT2i agents consisted of dapagliflozin, empagliflozin, ertugliflozin and sotagliflozin; the control agent was placebo across all the studies. Dapagliflozin was used as the intervention agent in 2 study, empagliflozin in 2, ertugliflozin in 1 and sotagliflozin in 2 studies (Table 1, Ref. [10, 11, 12, 16, 17, 18, 19]). The inclusion and exclusion criteria are displayed in the **Supplementary Materials**.

**Table 1.** **Baseline characteristics of included studies**.

Study ID	Registration number	Mean F-U period	SGLT2i	Control agent	Mean Age (years)	DM (%)	Systolic BP (mmHg)	Ejection fraction (%)	NT-proBNP (pg/mL)	ACEI/ARB (%)	β-blocker (%)
Kato *et al*. [16], 2019	NCT01730534	4.2 years	Dapagliflozin	Placebo	65	100	135 ± 15	55	NA	85	77
Savarese *et al*. [17], 2021	NCT01131676	3.1 years	Empagliflozin	Placebo	66	100	138 ± 16	NA	NA	74/61	56/67
Cosentino *et al*. [11], 2020	NCT01986881	3.5 years	Ertugliflozin	Placebo	64	100	134 ± 14	NA	NA	85	79
Anker *et al*. [10], 2021	NCT03057951	26 months	Empagliflozin	Placebo	72	49	132 ± 16	59	970	NA	NA
Bhatt *et al*. [18], 2021	NCT03521934	9 months	Sotagliflozin	Placebo	69	100	122	NA	1779	82	92
Bhatt *et al*. [19], 2021	NCT03315143	16 months	Sotagliflozin	Placebo	69	100	138	60	197	89	63
Solomon *et al*. [12], 2022	NCT03619213	2.3 years	Dapagliflozin	Placebo	72	45	128 ± 15	54	1011	72	76

The detail of included studies is displayed in the **Supplementary Materials**.

### 3.3 Primary Outcome

**Fig. 2.**
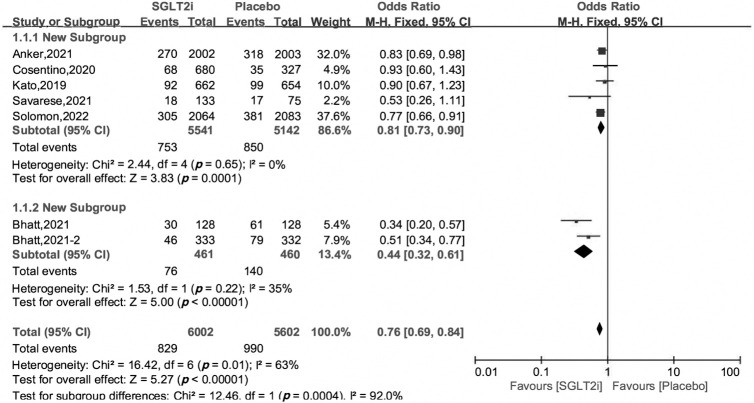
**Cardiovascular death and hospitalization for heart failure of SGLT2i *vs*. placebo in HFpEF patients**.

The primary outcome was a composite endpoint of cardiovascular death and hospitalization for heart failure (HHF). All the 7 included studies reported the primary outcome. Using the heterogeneity across trials, we divided the 7 RCTs into two subgroups, one subgroup consisted of the EMPEROR-Preserved [10], VERTIS CV [11], DECLARE-TIMI58 [16], DELIVER [12] and EMPA-REG OUTCOME trials [17], and another included the SOLOIST-WHF [18] and SCORED trials [19]. The OR and 95% CI were 0.81 [0.73, 0.90], 0.44 [0.32, 0.61], respectively, and the heterogeneity across trials was 0% and 35%. The overall OR and 95% CI were 0.76 [0.69, 0.84], and the overall heterogeneity was 63% (Fig. 2). The OR and 95% CI using random effects model were 0.71 [0.58, 0.86], *p* = 0.0007 (S4 in **Supplemental Materials**).

**Fig. 3.**
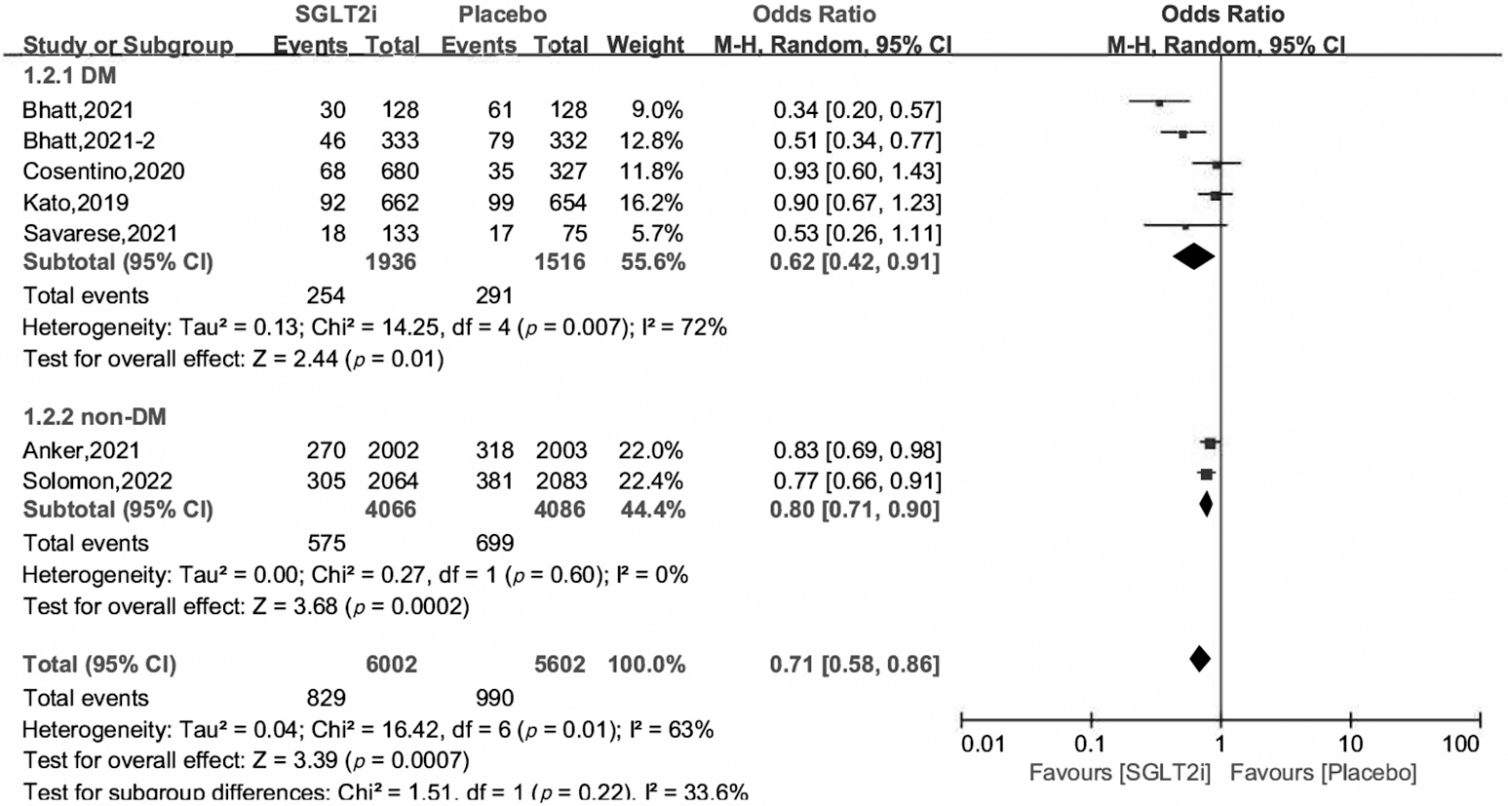
**Subgroup analysis of primary outcome by DM status**.

**Fig. 4.**
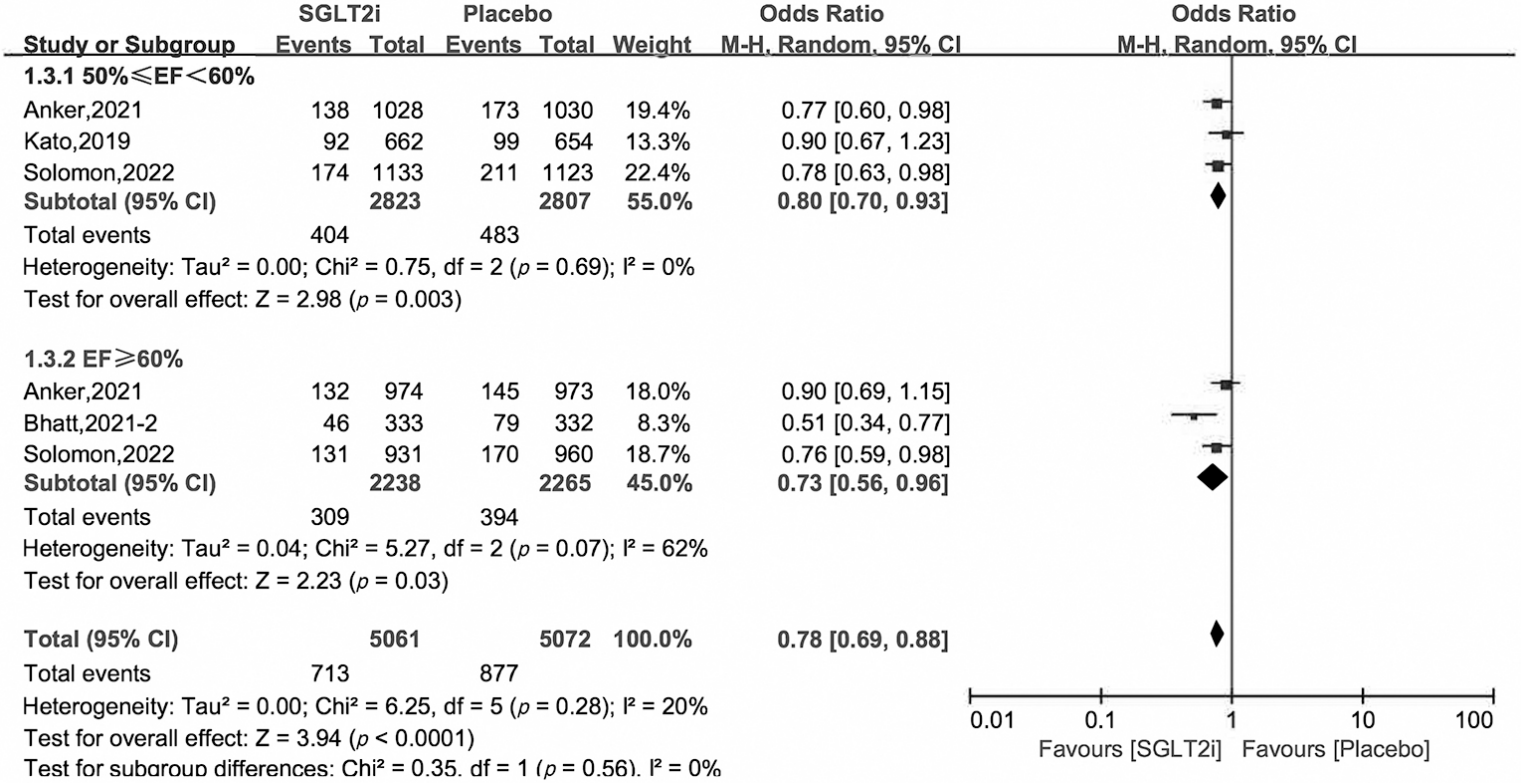
**Subgroup analysis of primary outcome by EF value**.

Subgroup analysis revealed that SGLT2i can reduce the incidence of the primary outcome by 20%–38%, irrespective of their DM status, with OR and 95% CI 0.62 [0.42, 0.91], 0.80 [0.71, 0.90], respectively. In the subgroup of 50% ≤ EF < 60%, the efficacy of SGLT2i was robust, with OR 0.80 [0.70, 0.93], and in patients with EF ≥60%, the benefit of SGLT2i still existed, OR 0.73 [0.56, 0.96], *p* = 0.03 (Figs. 3,4).

### 3.4 Secondary Outcomes

**Fig. 5.**
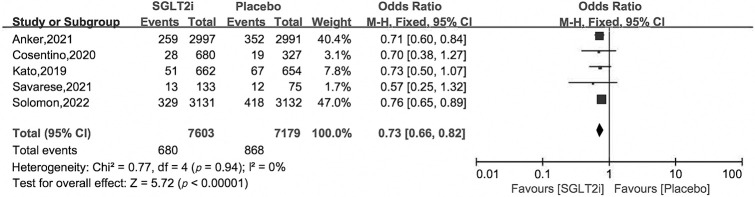
**Hospitalization for heart failure of SGLT2i *vs*. placebo in HFpEF patients**.

**Fig. 6.**
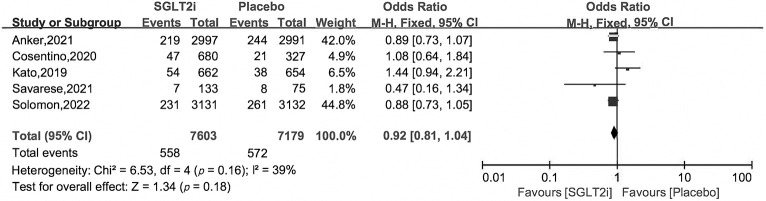
**Cardiovascular death of SGLT2i *vs*. placebo in HFpEF patients**.

**Fig. 7.**
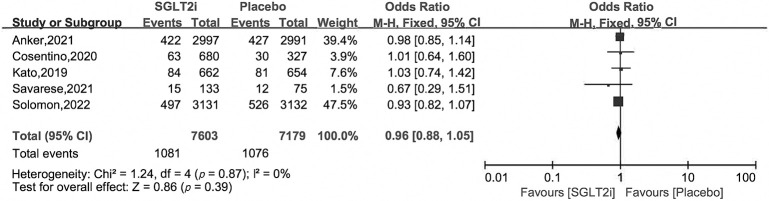
**All cause death of SGLT2i *vs*. placebo in HFpEF patients**.

Owing to the lack of data in the SOLOIST-WHF and SCORED studies [18, 19], we analyzed secondary outcomes of cardiovascular death, HHF and all cause death using data from DECLARE-TIMI58, EMPA-REG OUTCOME, DELIVER, EMPEROR-Preserved and VERTIS CV studies (for EMPEROR-Preserved and DELIVER, as there is no data of patients with EF ≥50%, we used data from those with EF ≥40%) [10, 11, 12, 16, 17]. A total of 14,782 patients were included in the subgroup analysis. Compared with placebo treatment, SGLT2i reduced HHF by 27% with OR and 95% CI 0.73 [0.66, 0.82], as for cardiovascular death and all cause death, there were no obvious differences between the two groups, with OR and 95% CI 0.92 [0.81, 1.04], 0.96 [0.88, 1.05] (Figs. 5,6,7).

### 3.5 Quality Assessment and Publication Bias

Overall, the quality of included studies was high. There was no obvious bias in random sequence generation, allocation concealment, blinding of participants and personnel, blinding of outcome assessment, incomplete outcome data and selective reporting (S1, **Supplemental Materials)**. In the funnel plot, we found no obvious publication bias (funnel plot in the S2, **Supplemental Materials**).

### 3.6 Sensitivity Analysis

As shown in S3 (**Supplemental Materials**), the influence analysis revealed that the primary outcome is robust. Irregardless of which study was omitted, the OR ranged from 0.65 to 0.87. The results remained unchanged whether a fixed or random effects model was used (random effects model in the **Supplemental Materials**). Estimated values of risk ratio (RR) and OR were also used to prove the efficacy of SGLT2i on HFpEF. The results remained unchanged whether or not the SOLOIST-WHF trial was omitted (**Supplemental Materials**).

## 4. Discussion

The efficacy of SGLT2i on HHF has been demonstrated in previous studies, which mainly focused on patients with atherosclerotic cardiovascular disease or other risk factors [20, 21, 22]. The EMPEROR-Reduced and DAPA-HF trials showed that SGLT2i could reduce the occurrence of the primary outcome of cardiovascular death and hospitalization for HF by 26%–30% in HFrEF patients [8]. Currently, only one study DELIVER proved the efficacy of SGLT2i on HFpEF [12]. Although the latest trial, the EMPEROR-Preserved study demonstrated the efficacy of SGLT2i in HF with EF ≥40%, it was mainly driven by patients in the subgroup of 40% ≤ EF < 50%, and the efficacy of SGLT2i on HFpEF with EF ≥60% was not confirmed. Our meta-analysis proved the efficacy of SGLT2i in HFpEF patients (both in EF ≥50% and EF ≥60%). We also found that SGLT2i reduced the incidence of HHF rather than death from cardiovascular or all-causes.

In a meta-analysis investigating effect of SGLT2i on cardiovascular outcomes in heart failure patients, Lu *et al*. [23] found that the effects of SGLT2i versus other treatment was significant in HFrEF but not statistically different in HFpEF. The small sample size may account for this, as there were only 2 studies included in the HFpEF subgroup [23]. Another real-world study revealed that initiation of SGLT2i could lower the risk of HHF or death by 45% in the subgroup of EF ≥50%, HR 0.55, 95% CI 0.43–0.70, but the population in the study did not include those with documented HF [24]. The EMPEROR-Preserved trial was the first large RCT to confirm that SGLT2i was effective in HF patients with EF ≥40% [10]. Two meta-analysis including HF patients with EF ≥40% confirmed this conclusion [25, 26]. However, HFpEF was defined as ejection fraction over 40% in the above studies. It has been shown that patients with 40% ≤ EF < 50% benefited most from empagliflozin, followed by those with an EF between 50% and 60%, and those with an ejection fraction greater than 60% benefited least in EMPEROR-Preserved study [10]. Currently, HFpEF is defined as an ejection fraction ≥50%, according to the 2021 ESC Heart Failure Guidelines [2]. There is sparse evidence of SGLT2i on HFpEF defined as ejection fraction over 50% besides EMPEROR-Preserved and DELIVER, especially in those HFpEF with an EF ≥60%. In our analysis, we included the latest EMPEROR-Preserved and DELIVER and found that SGLT2i could reduce the incidence of the primary outcome by approximately 24% compared with placebo (0.76 [0.69, 0.84]) in HFpEF patients. In the subgroup of EF ≥60%, the OR and 95% CI for the primary outcome were 0.73 [0.56, 0.96], *p* = 0.03, which was consistent with the results in the DELIVER trial [12].

In the analysis on primary outcome, the heterogeneity across trials was 69%, indicating substantial heterogeneity. Considering the random effect model may weaken the real effects of SGLT2i versus placebo, we used the fixed effect model and divided the 7 trials into 2 subgroups to eliminate the potential heterogeneity. The heterogeneity across trials decreased to 0% and 35% from 69%, and the OR and 95% CI were 0.81 [0.73, 0.90], 0.44 [0.32, 0.61], respectively. The random effect model was also performed to further verify the efficacy of SGLT2i, and the OR and 95% CI were 0.71 [0.58, 0.86], *p* = 0.0007 (S4, **Supplemental Materials**). We performed an influence analysis by omitting one study every time, and found that the OR of SGLT2i versus placebo ranged from 0.65 to 0.87. These analyses show that our results were reliable.

We also performed a subgroup analysis based on DM status and found that SGLT2i reduced the events of the primary outcome regardless of the DM status. This may be due to the mechanism of SGLT2i on the cardiovascular system. First, the benefit of SGLT2 inhibitors may be due to long-term changes in tissue sodium management after the initial diuresis, which lowers blood pressure, reduces ventricular afterload and reverses remodeling [27]. Second, overactivity of the sympathetic nervous system is another important cause of heart failure progression, and SGLT2i could reduce cardiac sympathetic activity [28]. Third, SGLT2i can increase ketone bodies in patients with or without type 2 diabetes mellitus (T2DM). Ketone bodies can improve cardiac energy metabolism in HF patients [27]; moreover, increased ketone bodies have been associated with lower sympathetic activity [29]. Fourth, the reduction in inflammation may play an important role in cardiovascular protection. Inflammation is an important contributor to heart failure severity regardless of the ejection fraction [30, 31]. SGLT2i have been shown to reduce inflammation in patients with diabetes [32]. The anti-inflammatory effect of SGLT2i may potentially decrease molecular processes related to inflammation, such as extracellular matrix turnover and fibrosis [33]. In addition, empagliflozin may also aid in cardio-protection by its effects on weight loss, glucose control, prevention of ischemia/reperfusion injury, decreasing epicardial fat mass, decreasing oxidative stress, delaying the progression of diabetic nephropathy, decreasing serum uric acid and reducing insulin resistance [34, 35].

Previous studies found that SGLT2i could reduce cardiovascular death or all-cause death only in HFrEF patients rather than HFpEF patients [9, 10, 36]. We found that SGLT2i just reduced the HHF in HFpEF patients, rather than the cardiovascular or all-cause death. Other studies have drawn similar conclusions [10, 37]. The improvements in symptoms may explain the reason why HHF rate was reduced in SGLT2i. In the PRESERVED-HF study, SGLT2i (dapagliflozin) improved Kansas City Cardiomyopathy Questionnaire (KCCQ) clinical summary scores, due to the improvements in both KCCQ total symptom scores and physical limitations scores [38]. The CHIEF-HF trial also demonstrated that SGLT2i (canagliflozin) significantly improved symptom burden in HF, regardless of EF or diabetes status [39]. These studies proved that SGLT2i were effective in improving symptoms, and the improvement in symptoms might reduce the incidence of HHF. The rate of cardiovascular deaths in the EMPEROR-Preserved trial was 3.4–3.8 events per 100 patient-year, but it was 7.6–8.1 events per 100 patient-year in the EMPEROR-Reduced study [10]. The mortality in HFpEF was lower than that in HFrEF, and the benefit of SGLT2i on cardiovascular death for HFpEF patients might also be decreased.

There are some limitations in our study. First, different studies used different cutoff values for the EFs (40%, 45%, 50%). While HFpEF has been defined with an EF over 50% according to the 2021 ESC Heart Failure Guidelines, the difference among studies can influence the outcomes [2]. Second, several baseline patient characteristics were unable to be derived in the studies which limited our ability to perform other subgroup analyses. Third, the SOLOIST-WHF study included patients with acute decompensated HF, although we excluded it and found that the results were still robust, it may introduce an element of selection bias. Fourth, the efficacy of SGLT2i on HFpEF is affected by DM status, however there was only two studies including non-DM patients. More studies on both patients with or without diabetes are needed. Fifth, some data in our meta-analysis was transformed from processed data, not from raw data, which may limit its accuracy. Even though we performed an influence analysis and verified the robustness of our results, it still could introduce bias. Finally, the inclusion criteria differed in each of the studies; and may also result in selection bias, which cannot be avoided by using statistical methodology.

## 5. Conclusions

In summary, our study proves the clinical efficacy of SGLT2i for treatment of HFpEF patients with or without diabetes, which was mainly driven by prevention of HHF rather than cardiovascular or all-cause death.

## Data Availability

All data generated or analyzed during this study are included in this published article.

## Abbreviations

HF, Heart failure; HFpEF, Heart failure with preserved ejection fraction; EF, Ejection fraction; HFrEF, Heart failure with reduced ejection fraction; HFmrEF, Heart failure with mildly reduced ejection fraction; SGLT2i, Sodium-glucose Cotransporter 2 Inhibitors; HR, Hazard ratio; CI, Confidence interval; RCT, Randomized controlled trial; KCCQ, Kansas City Cardiomyopathy Questionnaire.

## Supplementary Material

Supplementary material associated with this article can be found, in the online version, at https://doi.org/10.31083/j.rcm2311374.
