# Supplementary material for: Efficacy of Sodium-Glucose Cotransporter 2 Inhibitors in Heart Failure with a Preserved Ejection Fraction: A Meta-Analysis of Randomized Controlled Trials
Source: Rev Cardiovasc Med. 2022 Oct 31;23(11):374. doi: 10.31083/j.rcm2311374 (PMC11269072; doi:10.31083/j.rcm2311374)
Supplement: Supplementary file 1 [file 2153-8174-23-11-374-s1.zip › 2153-8174-23-11-374-s1/Supplementary Materials.pdf]

Supplementary Materials

Search Strategy

Pubmed

((((((((((((((("Sodium-Glucose Transporter 2 Inhibitors"[Mesh]) OR (Dapagliflozin[Title/Abstract])) OR (Canagliflozin[Title/Abstract])) OR (Empagliflozin[Title/Abstract])) OR (Ipragliflozin[Title/Abstract])) OR (Sergliflozin[Title/Abstract])) OR (Remogliflozin[Title/Abstract])) OR (Tofogliflozin[Title/Abstract])) OR (Luseogliflozin[Title/Abstract])) OR (Sotagliflozin[Title/Abstract])) OR (Ertugliflozin[Title/Abstract])) OR (Velagliflozin[Title/Abstract])) OR (Licogliflozin[Title/Abstract])) OR (Mizagliflozin[Title/Abstract])) AND ((Heart Failure[Title/Abstract]) OR ("Heart Failure"[Mesh]))) AND ((clinical[tiab] AND trial[tiab]) OR "clinical trials as topic"[mesh] OR "clinical trial"[pt] OR random*[tiab] OR "random allocation"[mesh] OR "therapeutic use"[sh]) ("Sodium-Glucose Transporter 2 Inhibitors"[MeSH Terms] OR "Dapagliflozin"[Title/Abstract] OR "Canagliflozin"[Title/Abstract] OR "Empagliflozin"[Title/Abstract] OR "Ipragliflozin"[Title/Abstract] OR "Sergliflozin"[Title/Abstract] OR "Remogliflozin"[Title/Abstract] OR "Tofogliflozin"[Title/Abstract] OR "Luseogliflozin"[Title/Abstract] OR "Sotagliflozin"[Title/Abstract] OR "Ertugliflozin"[Title/Abstract] OR "Velagliflozin"[Title/Abstract] OR "Licogliflozin"[Title/Abstract] OR "Mizagliflozin"[Title/Abstract]) AND ("Heart Failure"[Title/Abstract] OR "Heart Failure"[MeSH Terms]) AND (("clinical"[Title/Abstract] AND "trial"[Title/Abstract]) OR "clinical trials as topic"[MeSH Terms] OR "clinical trial"[Publication Type] OR "random*"[Title/Abstract] OR "random allocation"[MeSH Terms] OR "therapeutic use"[MeSH Subheading]). September,2022

Embase

No.	Query Results	Results	Date
#20.	#15 AND #18 AND #19	3,969	1 Sep 2022
#19.	'clinical':ti,ab AND 'trial':ti,ab OR 'clinical trial'/exp OR random* OR 'drug therapy':lnk	6,630,404	1 Sep 2022
#18.	#16 OR #17	662,105	1 Sep 2022
#17.	'heart failure':ab,ti	325,761	1 Sep 2022
#16.	'heart failure'/exp	611,023	1 Sep 2022
#15.	#1 OR #2 OR #3 OR #4 OR #5 OR #6 OR #7 OR #8 OR #9 OR #10 OR #11 OR #12 OR #13 OR #14	19,577	1 Sep 2022
#14.	mizagliflozin:ab,ti	12	1 Sep 2022
#13.	licogliflozin:ab,ti	16	1 Sep 2022
#12.	ertugliflozin:ab,ti	337	1 Sep 2022
#11.	velagliflozin:ab,ti	7	1 Sep 2022
#10.	sotagliflozin:ab,ti	209	1 Sep 2022
#9.	luseogliflozin:ab,ti	233	1 Sep 2022
#8.	tofogliflozin:ab,ti	229	1 Sep 2022
#7.	remogliflozin:ab,ti	91	1 Sep 2022
#6.	sergliflozin:ab,ti	17	1 Sep 2022

#5. ipragliflozin:ab,ti 405 1 Sep 2022

#4. empagliflozin:ab,ti 3,624 1 Sep 2022

#3. canagliflozin:ab,ti 2,383 1 Sep 2022

#2. dapagliflozin:ab,ti 3,643 1 Sep 2022

#1. 'sodium glucose cotransporter 2 inhibitor'/exp 19,302 1 Sep 2022

....

Cochrane

ID Search Hits

#1 MeSH descriptor: [Sodium-Glucose Transporter 2 Inhibitors] explode all trees 536

#2 (Dapagliflozin):ti,ab,kw 1538

#3 (Canagliflozin):ti,ab,kw 673

#4 (Empagliflozin):ti,ab,kw 1424

#5 (Ipragliflozin):ti,ab,kw 158

#6 (Sergliflozin):ti,ab,kw 2

#7 (Remogliflozin):ti,ab,kw30

#8 (Tofogliflozin):ti,ab,kw 89

#9 (Luseogliflozin):ti,ab,kw96

#10 (Luseogliflozin):ti,ab,kw96

#11 (Ertugliflozin):ti,ab,kw 167

#12 (Velagliflozin):ti,ab,kw 0

#13 (Licogliflozin):ti,ab,kw 14

#14 (Mizagliflozin):ti,ab,kw 3

#15 #1 or #2 or #3 or #4 or #5 or #6 or #7 or #8 3796

#16 #9 or #10 or #11 or #12 or #13 or #14 or #154018

#17 MeSH descriptor: [Heart Failure] explode all trees 10538

#18 #16 and #17 214

S1: Risk of bias

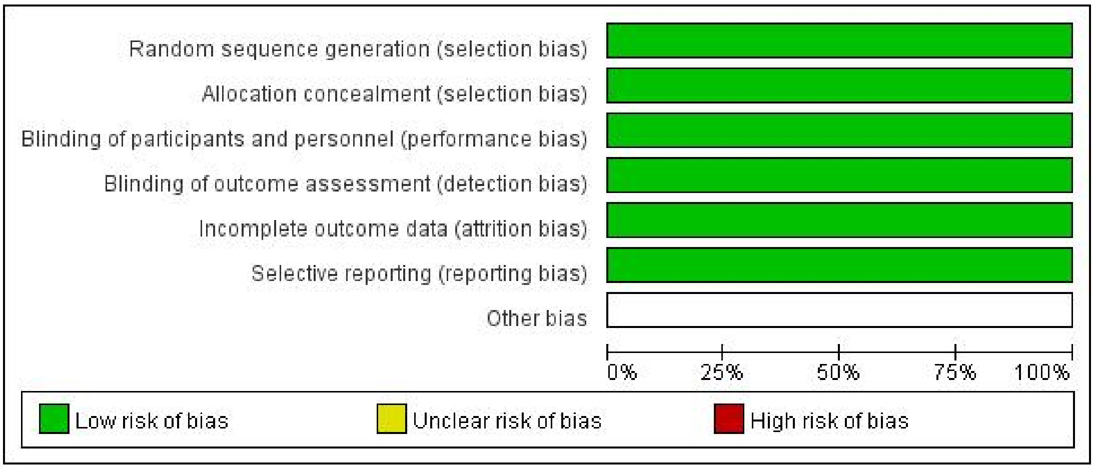

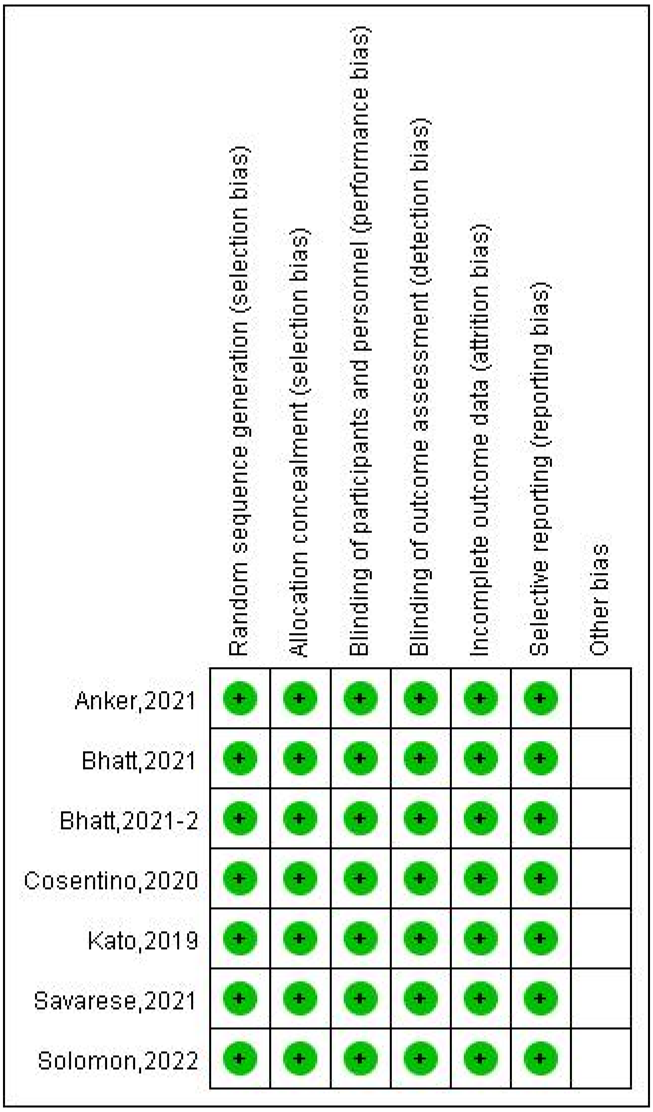

S2: Funnel plot

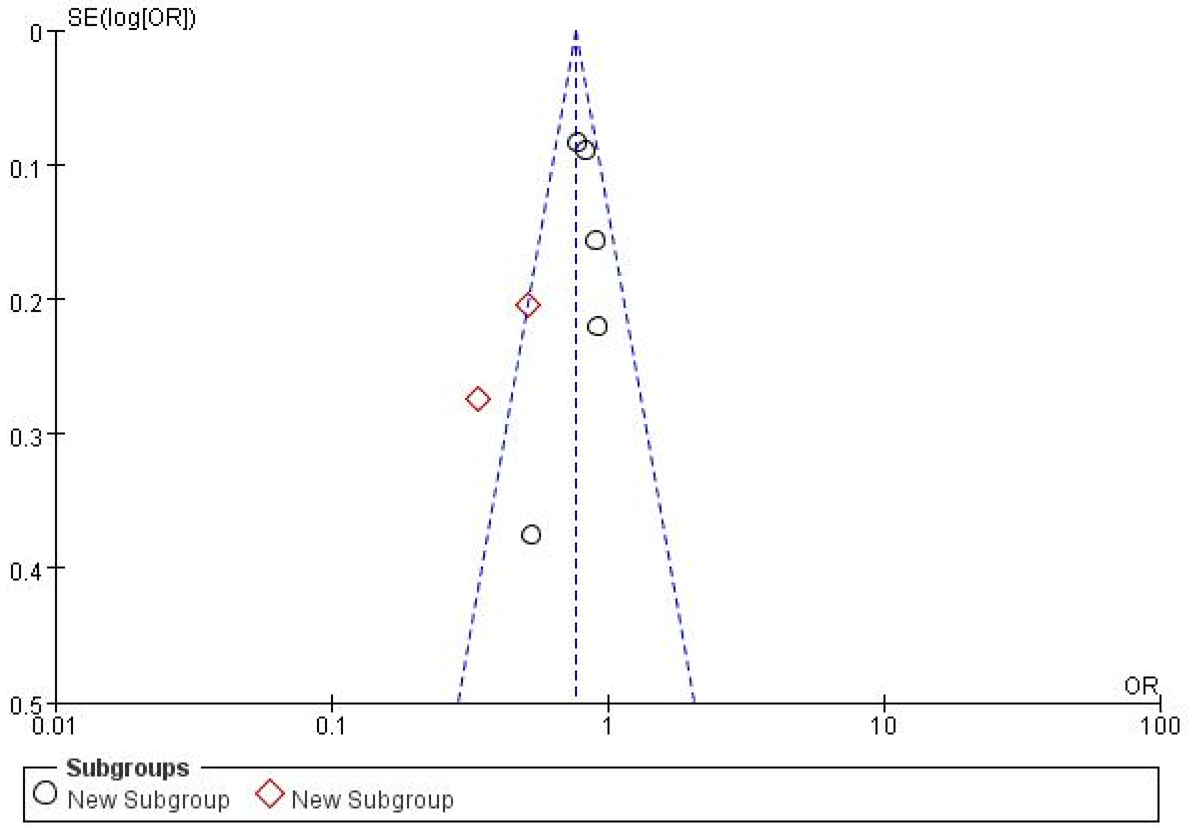

S3: Influence analysis

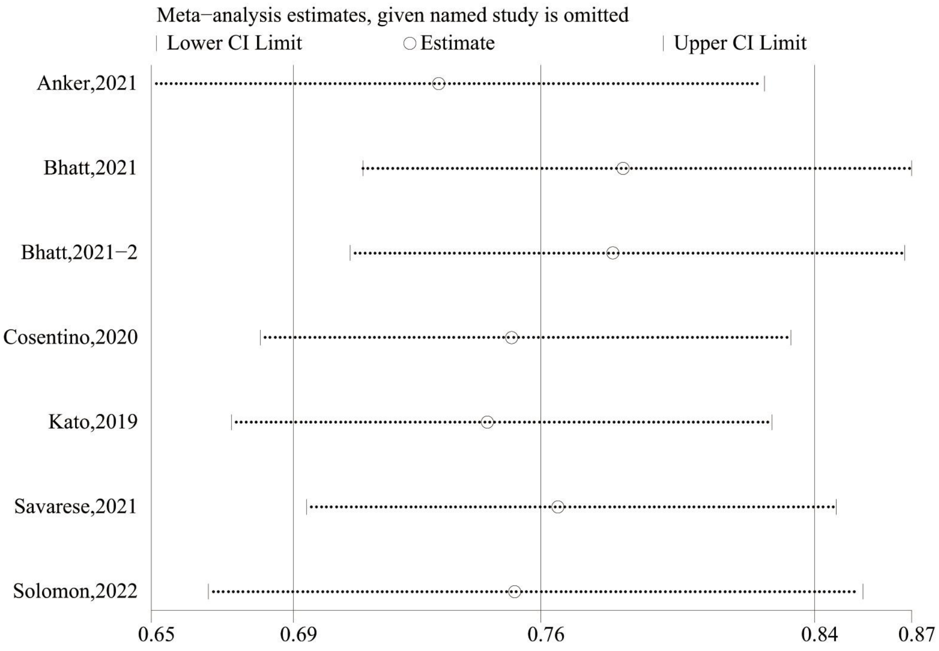

S4: Cardiovascular death or hospitalization for heart failure of SGLT2i vs. placebo in HFpEF patients using random effect model

Study or Subgroup	SGLT2i Events	SGLT2i Total	Placebo Events	Placebo Total	Weight	Odds Ratio M-H, Random, 95% CI
**1.5.1 New Subgroup**						
Anker,2021	270	2002	318	2003	22.0%	0.83 [0.69, 0.98]
Bhatt,2021	30	128	61	128	9.0%	0.34 [0.20, 0.57]
Bhatt,2021-2	46	333	79	332	12.8%	0.51 [0.34, 0.77]
Cosentino,2020	68	680	35	327	11.8%	0.93 [0.60, 1.43]
Kato,2019	92	662	99	654	16.2%	0.90 [0.67, 1.23]
Savarese,2021	18	133	17	75	5.7%	0.53 [0.26, 1.11]
Solomon,2022	305	2064	381	2083	22.4%	0.77 [0.66, 0.91]
**Subtotal (95% CI)**		**6002**		**5602**	**100.0%**	**0.71 [0.58, 0.86]**
Total events	829		990			
Heterogeneity: Tau² = 0.04; Chi² = 16.42, df = 6 (P = 0.01); I² = 63%						
Test for overall effect: Z = 3.39 (P = 0.0007)						
**Total (95% CI)**		**6002**		**5602**	**100.0%**	**0.71 [0.58, 0.86]**
Total events	829		990			
Heterogeneity: Tau² = 0.04; Chi² = 16.42, df = 6 (P = 0.01); I² = 63%						
Test for overall effect: Z = 3.39 (P = 0.0007)						
Test for subgroup differences: Not applicable						

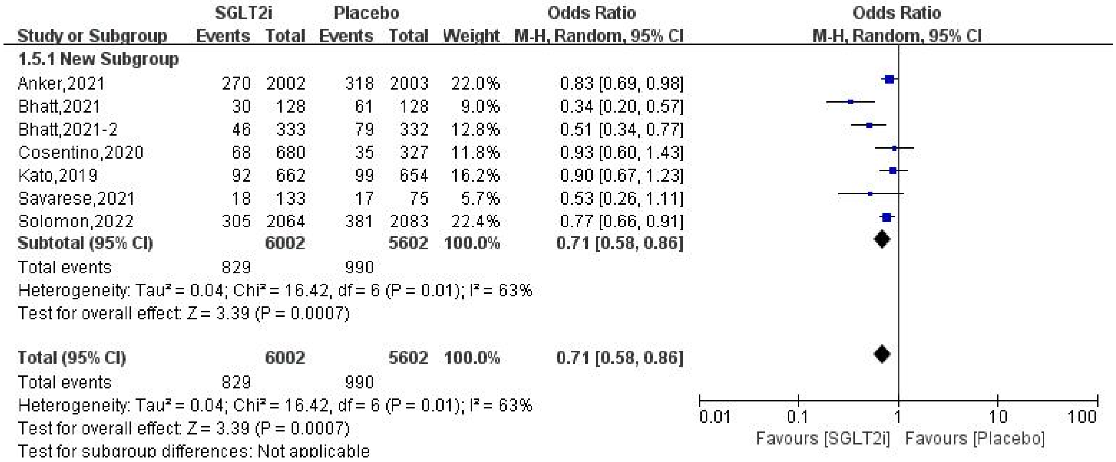

Cardiovascular death of SGLT2i vs. placebo in HFpEF patients using random effect model

Study or Subgroup	SGLT2i Events	SGLT2i Total	Placebo Events	Placebo Total	Weight	Odds Ratio M-H, Random, 95% CI
Anker,2021	219	2997	244	2991	36.0%	0.89 [0.73, 1.07]
Cosentino,2020	47	680	21	327	10.1%	1.08 [0.64, 1.84]
Kato,2019	54	662	38	654	14.2%	1.44 [0.94, 2.21]
Savarese,2021	7	133	8	75	2.9%	0.47 [0.16, 1.34]
Solomon,2022	231	3131	261	3132	36.8%	0.88 [0.73, 1.05]
**Total (95% CI)**		**7603**		**7179**	**100.0%**	**0.95 [0.79, 1.14]**
Total events	558		572			
Heterogeneity: Tau² = 0.02; Chi² = 6.53, df = 4 (P = 0.16); I² = 39%						
Test for overall effect: Z = 0.57 (P = 0.57)						

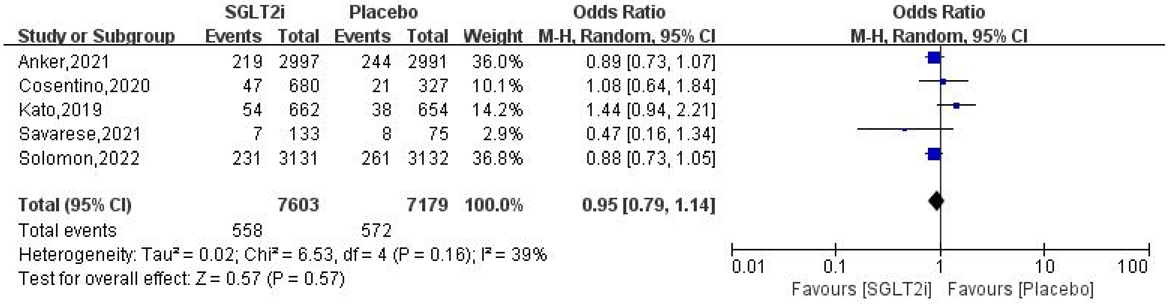

Hospitalization for heart failure of SGLT2i vs. placebo in HFpEF patients using random effect model

Study or Subgroup	SGLT2i Events	SGLT2i Total	Placebo Events	Placebo Total	Weight	Odds Ratio M-H, Random, 95% CI
Solomon,2022	329	3131	418	3132	47.9%	0.76 [0.65, 0.89]
Savarese,2021	13	133	12	75	1.6%	0.57 [0.25, 1.32]
Kato,2019	51	662	67	654	7.8%	0.73 [0.50, 1.07]
Cosentino,2020	28	680	19	327	3.2%	0.70 [0.38, 1.27]
Anker,2021	259	2997	352	2991	39.6%	0.71 [0.60, 0.84]
**Total (95% CI)**		**7603**		**7179**	**100.0%**	**0.73 [0.66, 0.82]**
Total events	680		868			
Heterogeneity: Tau² = 0.00; Chi² = 0.77, df = 4 (P = 0.94); I² = 0%						
Test for overall effect: Z = 5.72 (P < 0.00001)						

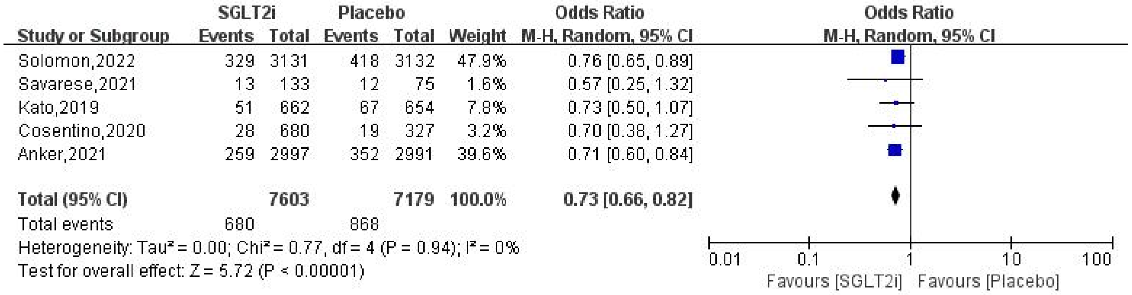

All cause death of SGLT2i vs. placebo in HFpEF patients using random effect model

Study or Subgroup	SGLT2i Events	SGLT2i Total	Placebo Events	Placebo Total	Weight	Odds Ratio M-H, Random, 95% CI
Anker,2021	422	2997	427	2991	39.9%	0.98 [0.85, 1.14]
Cosentino,2020	63	680	30	327	4.0%	1.01 [0.64, 1.60]
Kato,2019	84	662	81	654	7.9%	1.03 [0.74, 1.42]
Savarese,2021	15	133	12	75	1.3%	0.67 [0.29, 1.51]
Solomon,2022	497	3131	526	3132	46.9%	0.93 [0.82, 1.07]
**Total (95% CI)**		**7603**		**7179**	**100.0%**	**0.96 [0.88, 1.05]**
Total events	1081		1076			
Heterogeneity: Tau² = 0.00; Chi² = 1.24, df = 4 (P = 0.87); I² = 0%						
Test for overall effect: Z = 0.86 (P = 0.39)						

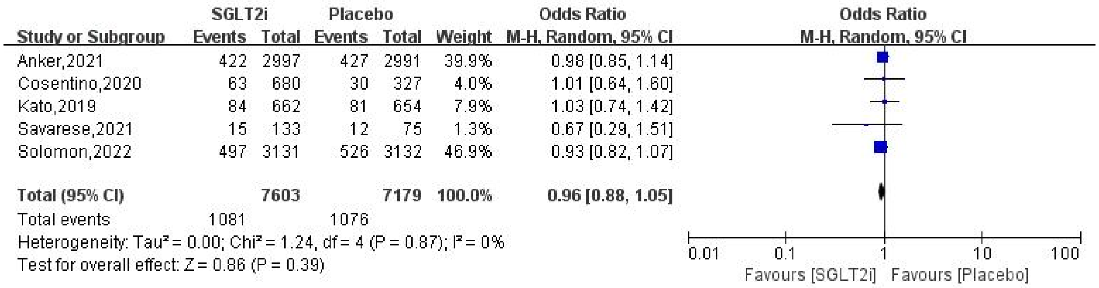

S5: Subgroup anslysis based on EF value using fixed effect model

Study or Subgroup	SGLT2i Events	SGLT2i Total	Placebo Events	Placebo Total	Weight	Odds Ratio M-H, Fixed, 95% CI
**1.3.1 50%≤EF<60%**						
Anker,2021	138	1028	173	1030	19.9%	0.77 [0.60, 0.98]
Kato,2019	92	662	99	654	11.4%	0.90 [0.67, 1.23]
Solomon,2022	174	1133	211	1123	23.8%	0.78 [0.63, 0.98]
**Subtotal (95% CI)**		**2823**		**2807**	**55.1%**	**0.80 [0.70, 0.93]**
Total events	404		483			
Heterogeneity: Chi² = 0.75, df = 2 (P = 0.69); I² = 0%						
Test for overall effect: Z = 2.98 (P = 0.003)						
**1.3.2 EF≥60%**						
Anker,2021	132	974	145	973	16.7%	0.90 [0.69, 1.15]
Bhatt,2021-2	46	333	79	332	9.1%	0.51 [0.34, 0.77]
Solomon,2022	131	931	170	960	19.1%	0.76 [0.59, 0.98]
**Subtotal (95% CI)**		**2238**		**2265**	**44.9%**	**0.76 [0.65, 0.89]**
Total events	309		394			
Heterogeneity: Chi² = 5.27, df = 2 (P = 0.07); I² = 62%						
Test for overall effect: Z = 3.31 (P = 0.0009)						
**Total (95% CI)**		**5061**		**5072**	**100.0%**	**0.78 [0.70, 0.87]**
Total events	713		877			
Heterogeneity: Chi² = 6.25, df = 5 (P = 0.28); I² = 20%						
Test for overall effect: Z = 4.43 (P < 0.00001)						
Test for subgroup differences: Chi² = 0.24, df = 1 (P = 0.62), I² = 0%						

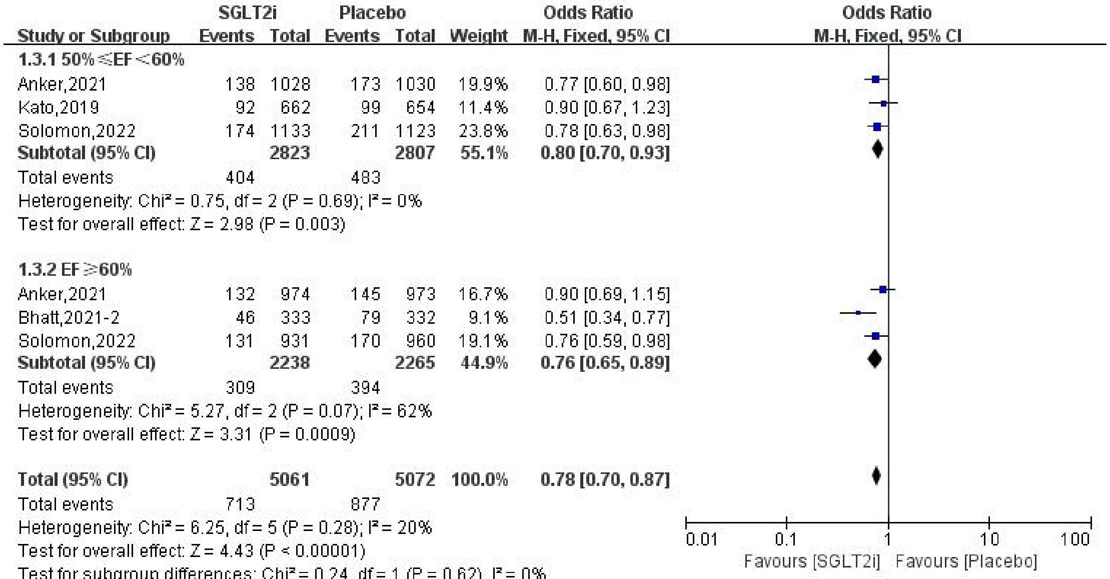

Primary outcome omitting SOLOIST-WHF trial

Study or Subgroup	SGLT2i Events	SGLT2i Total	Placebo Events	Placebo Total	Weight	Odds Ratio M-H, Random, 95% CI
**1.5.1 New Subgroup**						
Anker,2021	270	2002	318	2003	30.4%	0.83 [0.69, 0.98]
Bhatt,2021	30	128	61	128	0.0%	0.34 [0.20, 0.57]
Bhatt,2021-2	46	333	79	332	9.9%	0.51 [0.34, 0.77]
Cosentino,2020	68	680	35	327	8.8%	0.93 [0.60, 1.43]
Kato,2019	92	662	99	654	15.2%	0.90 [0.67, 1.23]
Savarese,2021	18	133	17	75	3.4%	0.53 [0.26, 1.11]
Solomon,2022	305	2064	381	2083	32.3%	0.77 [0.66, 0.91]
**Subtotal (95% CI)**		**5874**		**5474**	**100.0%**	**0.78 [0.68, 0.89]**
Total events	799		929			
Heterogeneity: Tau² = 0.01; Chi² = 7.11, df = 5 (P = 0.21); I² = 30%						
Test for overall effect: Z = 3.54 (P = 0.0004)						
**Total (95% CI)**		**5874**		**5474**	**100.0%**	**0.78 [0.68, 0.89]**
Total events	799		929			
Heterogeneity: Tau² = 0.01; Chi² = 7.11, df = 5 (P = 0.21); I² = 30%						
Test for overall effect: Z = 3.54 (P = 0.0004)						
Test for subgroup differences: Not applicable						

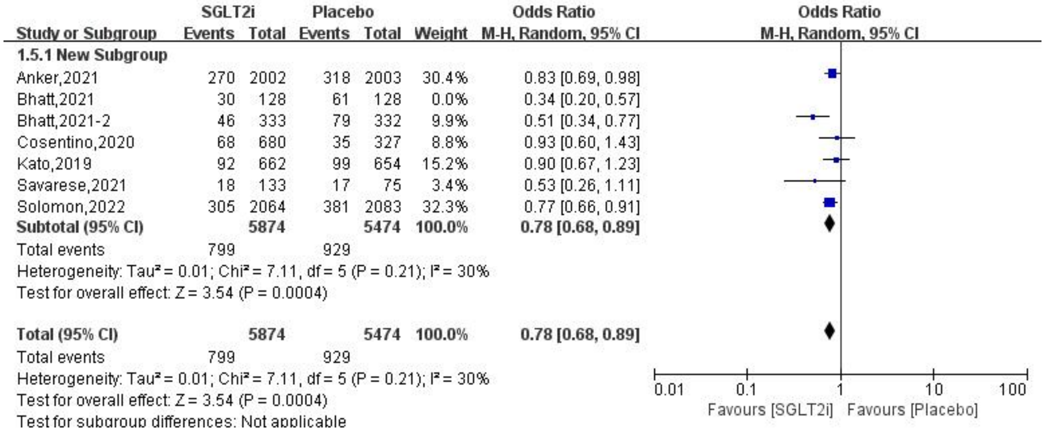

Cardiovascular death and hospitalization for heart failure of SGLT2i vs. placebo using RR and 95% CI

Study or Subgroup	SGLT2i Events	SGLT2i Total	Placebo Events	Placebo Total	Weight	Risk Ratio M-H, Fixed, 95% CI
**1.5.1 New Subgroup**						
Anker,2021	270	2002	318	2003	31.6%	0.85 [0.73, 0.99]
Bhatt,2021	30	128	61	128	6.1%	0.49 [0.34, 0.71]
Bhatt,2021-2	46	333	79	332	7.9%	0.58 [0.42, 0.81]
Cosentino,2020	68	680	35	327	4.7%	0.93 [0.64, 1.37]
Kato,2019	92	662	99	654	9.9%	0.92 [0.71, 1.19]
Savarese,2021	18	133	17	75	2.2%	0.60 [0.33, 1.09]
Solomon,2022	305	2064	381	2083	37.7%	0.81 [0.70, 0.93]
**Subtotal (95% CI)**		**6002**		**5602**	**100.0%**	**0.80 [0.73, 0.87]**
Total events	829		990			
Heterogeneity: Chi² = 13.76, df = 6 (P = 0.03); I² = 56%						
Test for overall effect: Z = 5.26 (P < 0.00001)						
**Total (95% CI)**		**6002**		**5602**	**100.0%**	**0.80 [0.73, 0.87]**
Total events	829		990			
Heterogeneity: Chi² = 13.76, df = 6 (P = 0.03); I² = 56%						
Test for overall effect: Z = 5.26 (P < 0.00001)						
Test for subgroup differences: Not applicable						

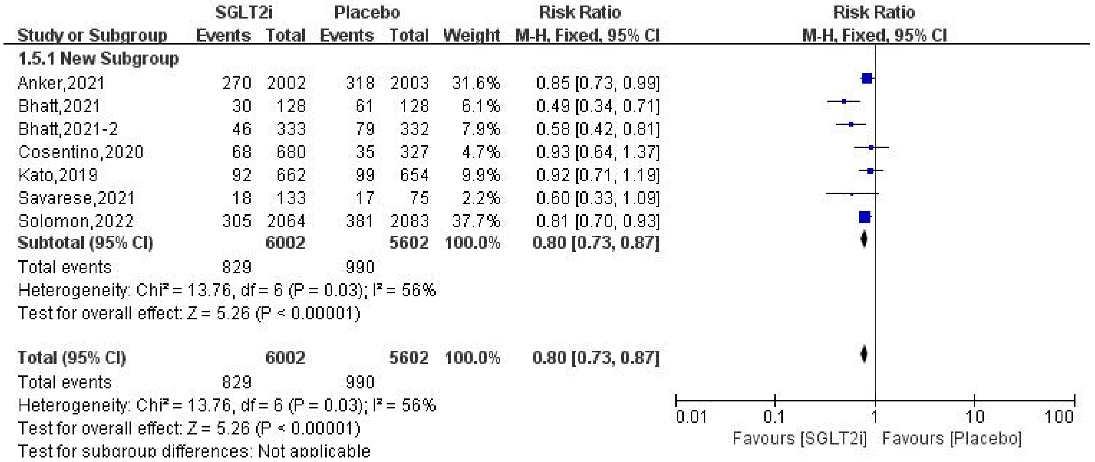

Cardiovascular death of SGLT2i vs. placebo using RR and 95% CI

Study or Subgroup	SGLT2i Events	SGLT2i Total	Placebo Events	Placebo Total	Weight	Risk Ratio M-H, Fixed, 95% CI
Anker,2021	219	2997	244	2991	42.0%	0.90 [0.75, 1.07]
Cosentino,2020	47	680	21	327	4.9%	1.08 [0.65, 1.77]
Kato,2019	54	662	38	654	6.6%	1.40 [0.94, 2.10]
Savarese,2021	7	133	8	75	1.8%	0.49 [0.19, 1.31]
Solomon,2022	231	3131	261	3132	44.8%	0.89 [0.75, 1.05]
**Total (95% CI)**		**7603**		**7179**	**100.0%**	**0.93 [0.83, 1.04]**
Total events	558		572			

Heterogeneity: $Chi^2 = 6.50$, df = 4 (P = 0.16); $I^2 = 39\%$
Test for overall effect: Z = 1.34 (P = 0.18)

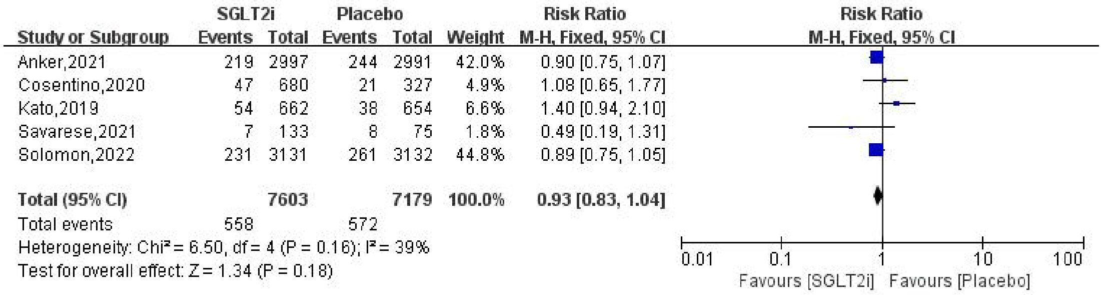

Hospitalization for heart failure of SGLT2i vs. placebo using RR and 95% CI

Study or Subgroup	SGLT2i Events	SGLT2i Total	Placebo Events	Placebo Total	Weight	Risk Ratio M-H, Fixed, 95% CI
Solomon,2022	329	3131	418	3132	47.6%	0.79 [0.69, 0.90]
Savarese,2021	13	133	12	75	1.7%	0.61 [0.29, 1.27]
Kato,2019	51	662	67	654	7.7%	0.75 [0.53, 1.06]
Cosentino,2020	28	680	19	327	2.9%	0.71 [0.40, 1.25]
Anker,2021	259	2997	352	2991	40.1%	0.73 [0.63, 0.86]
**Total (95% CI)**		**7603**		**7179**	**100.0%**	**0.76 [0.69, 0.83]**
Total events	680		868			

Heterogeneity: $Chi^2 = 0.86$, df = 4 (P = 0.93); $I^2 = 0\%$
Test for overall effect: Z = 5.71 (P < 0.00001)

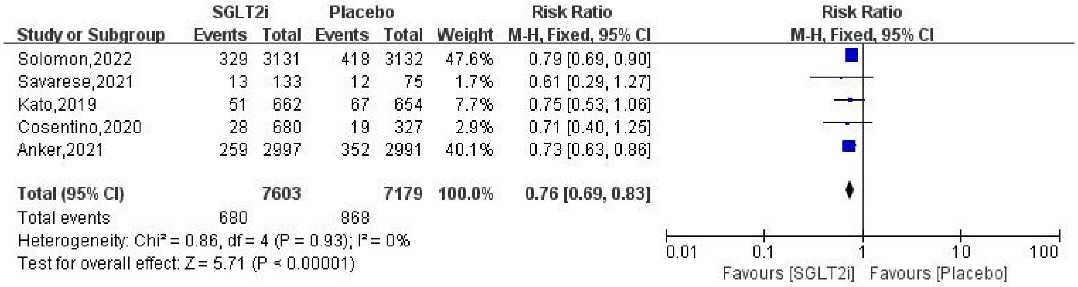

All cause death of SGLT2i vs. placebo using RR and 95% CI

Study or Subgroup	SGLT2i Events	SGLT2i Total	Placebo Events	Placebo Total	Weight	Risk Ratio M-H, Fixed, 95% CI
Anker,2021	422	2997	427	2991	39.2%	0.99 [0.87, 1.12]
Cosentino,2020	63	680	30	327	3.7%	1.01 [0.67, 1.53]
Kato,2019	84	662	81	654	7.5%	1.02 [0.77, 1.36]
Savarese,2021	15	133	12	75	1.4%	0.70 [0.35, 1.43]
Solomon,2022	497	3131	526	3132	48.2%	0.95 [0.84, 1.06]
**Total (95% CI)**		**7603**		**7179**	**100.0%**	**0.97 [0.89, 1.04]**
Total events	1081		1076			

Heterogeneity: $Chi^2 = 1.23$, df = 4 (P = 0.87); $I^2 = 0\%$
Test for overall effect: Z = 0.86 (P = 0.39)

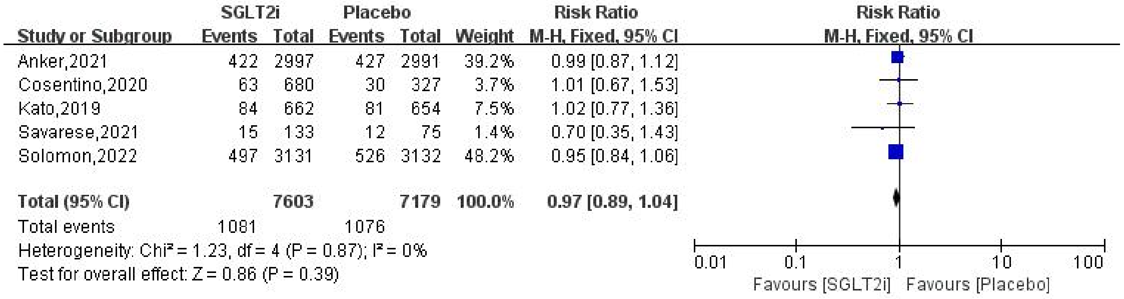

Inclusion criteria and exclusion criteria

Kato,2019

Inclusion criteria

1. Provision of informed consent prior to any study specific procedures
2. Female or male aged ≥40 years
3. Diagnosed with Type 2 Diabetes
4. High Risk for Cardiovascular events

Exclusion criteria

1. Diagnosis of Type 1 diabetes mellitus History of bladder cancer or history of radiation therapy to the lower abdomen or pelvis at any time
2. Chronic cystitis and/or recurrent urinary tract infections
3. Pregnant or breast-feeding patients

Savarese,2021

Inclusion criteria

1. Diagnosis of type 2 diabetes mellitus prior to informed consent
2. Male or female patients on diet and exercise regimen who are drug naive or pre treated with any background therapy. Antidiabetic therapy has to be unchanged for 12 weeks prior to randomization.
3. Glycosylated haemoglobin (HbA1c) of >= 7.0% and <=10% for patients on background therapy or HbA1c >= 7.0% and <= 9.0% for drug naive patients
4. Age >= 18 years
5. Body Mass index <= 45 at Visit 1
6. Signed and dated informed consent
7. High cardiovascular risk

Exclusion criteria

1. Uncontrolled hyperglycaemia with a glucose level >240 mg/dl (>13.3 mmol/L) after an overnight fast during placebo run-in and confirmed by a second measurement (not on the same day)
2. Indication of liver disease, defined by serum levels of either alanine aminotransferase (ALT), aspartate aminotransferase ALT or alkaline phosphatase above 3 x upper limit of normal (ULN) as determined at screening and/or run in.
3. Planned cardiac surgery or angioplasty within 3 months
4. Impaired renal function, defined as Glomerular Filtration Rate <30 ml/min (severe renal impairment, Modification of Diet in Renal Disease formula) during screening or run in.
5. Bariatric surgery within the past two years and other gastrointestinal surgeries that induce chronic malabsorption
6. Blood dyscrasias or any disorders causing haemolysis or unstable Red Blood Cell (e.g. malaria, babesiosis, haemolytic anemia)
7. Medical history of cancer (except for basal cell carcinoma) and/or treatment for cancer within the last 5 years
8. Contraindications to background therapy according to the local label

9. Treatment with anti-obesity drugs (e.g. sibutramine, orlistat) 3 months prior to informed consent or any other treatment at the time of screening (i.e. surgery, aggressive diet regimen, etc.) leading to unstable body weight
10. Current treatment with systemic steroids at time of informed consent or change in dosage of thyroid hormones within 6 weeks prior to informed consent or any other uncontrolled endocrine disorder except type 2 diabetes mellitus
11. Pre-menopausal women (last menstruation <+ 1 year prior to informed consent) who:are nursing or pregnant or are of child-bearing potential and are not practicing an acceptable method of birth control, or do not plan to continue using this method throughout the study and do not agree to submit to periodic pregnancy testing during participation in the trial. Acceptable methods of birth control include tubal ligation, transdermal patch, intra uterine devices/systems, oral, implantable or injectable contraceptives, sexual abstinence, double barrier method and vasectomised partner
12. Alcohol or drug abuse within the 3 months prior to informed consent that would interfere with trial participation or any ongoing condition leading to a decreased compliance to study procedures or study drug intake
13. Participation in another trial with an investigational drug within 30 days prior to informed consent
14. Any other clinical condition that would jeopardize patients safety while participating in this clinical trial
15. Acute coronary syndrome, stroke or TIA within 2 months prior to informed consent

Cosentino,2020
Inclusion criteria
1. Diagnosis of T2DM in accordance with American Diabetes Association (ADA) guidelines
2. Hemoglobin A1c (A1C) at the start of study participation of 7.0-10.5% (53-91 mmol/mol)
3. On stable allowable anti-hyperglycemic agents (AHA) or on no background AHA for at least 8 weeks prior to the study participation
4. Body Mass Index (BMI) > or = to 18.0 kg/m^2
5. Evidence or a history of atherosclerosis involving the coronary, cerebral or peripheral vascular systems
6. There is adequate documentation of the objective evidence that the participant has established vascular disease such as investigational site's medical records, copies of such records from other institutions, or a letter from a referring physician that specifically states the diagnosis and date of the most recent occurrence of the qualifying event(s) or procedure(s).
7. Male, female not or reproductive potential, or female of reproductive potential who agrees to be abstinent from heterosexual activity or agrees to use or have their partner use 2 acceptable methods of contraception

Exclusion criteria

1. Previous randomization into this trial
2. Experiencing a cardiovascular event (myocardial infarction or stroke) or undergoing coronary angioplasty or peripheral intervention procedure between the Screening Visit and randomization
3. Undergoing any cardiovascular surgery (valvular surgery) within 3 months of study participation
4. Planned revascularization or peripheral intervention procedure or other cardiovascular surgery
5. New York Heart Association (NYHA) IV heart failure at study participation
6. History of type 1 diabetes mellitus or a history of ketoacidosis

Anker,2021

Inclusion criteria

1. Male or female patient, age >= 18 years at screening. For Japan only: Age >=20 years at screening
2. Patients with chronic HF (Chronic Heart Failure) NYHA (New York Heart Association classification) class II-IV and preserved EF (Ejection Fraction)(LVEF (Left Ventricular Ejection Fraction) > 40 %) and elevated NT-proBNP (N-terminal of the prohormone brain natriuretic peptide) > 300 pg/ml for patients without AF, OR > 900 pg/ml for patients with AF, analysed at the Central laboratory at Visit 1
3. Structural heart disease within 6 months prior to Visit 1, OR documented HHF (Hospitalisation for Heart Failure) within 12 months prior to Visit 1
4. Stable dose of oral diuretics, if prescribed
5. Signed and dated written ICF (informed consent form)
6. Further inclusion criteria apply

Exclusion criteria

1. Myocardial infarction, coronary artery bypass graft surgery or other major cardiovascular surgery, stroke or TIA (Transient Ischaemic Attack) in past 90 days prior to Visit 1
2. Heart transplant recipient or listed for heart transplant
3. Acute decompensated HF (Heart Failure)
4. Systolic blood pressure (SBP) >= 180 mmHg at Visit 2.
5. Symptomatic hypotension and/or a SBP < 100 mmHg
6. Indication of liver disease,
7. Impaired renal function, defined as eGFR (Estimated Glomerular Filtration Rate) < 20 mL/min/1.73 m2 (CKD-EPI (Chronic Kidney Disease Epidemiology Collaboration Equation))cr or requiring dialysis
8. History of ketoacidosis
9. Current use or prior use of a SGLT (Sodium-glucose co-transporter) -2 inhibitor or combined SGLT-1 and 2 inhibitor
10. Currently enrolled in another investigational device or drug trial
11. Known allergy or hypersensitivity to empagliflozin or other SGLT-2 inhibitors

12. Women who are pregnant, nursing, or who plan to become pregnant while in the trial
13. Further exclusion criteria may apply

Bhatt,2021

Inclusion criteria

1. Type 2 Diabetes Mellitus.
2. Admitted to the hospital, or urgent heart failure visit for worsening heart failure.
3. Prior diagnosis of heart failure (> 3 months).
4. Prior chronic treatment for heart failure with a loop diuretic (eg furosemide, torsemide, bumetanide) for > 30 days.
5. Randomized when hemodynamically stable, prior to hospital discharge or within 3 days of discharge.
6. Brain natriuretic peptide (BNP) ≥150 pg/mL (≥450 pg/mL for patients with atrial fibrillation) or N-terminal B-type natriuretic peptide ≥600 pg/mL (≥1800 pg/mL for patients with atrial fibrillation).
7. Patients with Left Ventricular Ejection Fraction <40% should be on beta-blockers and renin-angiotensin-aldosterone system (RAAS) inhibitors as per local guidelines unless contraindicated.
8. Signed written informed consent.

Exclusion criteria

1. Age < 18 years or > 85 years.
2. Worsening heart failure attributed to other causes such as pulmonary embolism, stroke, heart attack.
3. Cardiac surgery or coronary procedure within 1 month or planned during study.
4. Lower extremity complications (such as skin ulcer, infection, osteomyelitis, and gangrene) identified during screening and requiring treatment at randomization.
5. Planning to start a sodium-glucose linked transporter-2 (SGLT2) inhibitor during the study.
6. Acute coronary syndromes within 3 months prior to Randomization.
7. Hemodynamically significant uncorrected primary valvular disease.
8. Significant pulmonary disease contributing substantially to the patient's dyspnea.
9. End stage Heart Failure.
10. History of diabetic ketoacidosis (DKA) or nonketotic hyperosmolar coma within 3 months prior to screening.
11. History of stroke within 3 months prior to randomization.
12. History of dialysis within 1 year prior to randomization.
13. History of solid organ transplant or on a transplant list (if heart transplant, defined as status 1 transplant).
14. Severe kidney disease as defined by glomerular filtration rate (eGFR) <30 mL/min/1.73 m^2.
15. Pregnancy.

Bhatt,2021-2

Inclusion criteria

1. Type 2 Diabetes Mellitus with glycosylated hemoglobin (HbA1c) ≥ 7%.
2. Estimated glomerular filtration rate (eGFR) ≥ 25 and ≤ 60 mL/min/1.73 m2.
3. Age 18 years or older with at least one major cardiovascular risk factor or age 55 years or older with at least two minor cardiovascular risk factors.
4. Signed written informed consent.

Exclusion criteria

1. Antihyperglycemic treatment has not been stable within 12 weeks prior to screening.
2. Planned coronary procedure or surgery after randomization.
3. Lower extremity complications (such as skin ulcer, infection, osteomyelitis, and gangrene) identified during screening and requiring treatment at randomization.
4. Planning to start a sodium-glucose linked transporter-2 (SGLT2) inhibitor during the study.

Solomon,2022

Inclusion criteria

1. Provision of signed informed consent prior to any study specific procedures.
2. Male or female patients age ≥40 years.
3. Documented diagnosis of symptomatic heart failure (NYHA class II-IV) at enrolment, and a medical history of typical symptoms/signs of heart failure ≥6 weeks before enrolment with at least intermittent need for diuretic treatment.
4. Left Ventricular Ejection Fraction (LVEF) >40% and evidence of structural heart disease (i.e. left ventricular hypertrophy or left atrial enlargement) documented by the most recent echocardiogram, and/or cardiac MR within the last 12 months prior to enrolment. For patients with prior acute cardiac events or procedures that may reduce LVEF, e.g. as defined in exclusion criterion 6, qualifying cardiac imaging assessment at least 12 weeks following the procedure/event is required.
5. Elevated NT-pro BNP levels.
6. Both ambulatory and hospitalised patients may be enrolled and randomised. Patients currently hospitalised for HF, must be off intravenous HF medications for at least 24 before randomisation.

Exclusion criteria

1. Receiving therapy with an SGLT2 inhibitor within 4 weeks prior to randomisation or previous intolerance to an SGLT2 inhibitor.
2. Type 1 diabetes mellitus (T1D).
3. eGFR <25 mL/min/1.73 m2 (CKD-EPI formula) at Visit 1.
4. Systolic blood pressure (BP) <95 mmHg on 2 consecutive measurements at 5-minute intervals, at Visit 1 or at Visit 2.
5. Systolic BP≥160 mmHg if not on treatment with ≥3 blood pressure lowering medications or ≥180 mmHg irrespective of treatments, on 2 consecutive measurements at 5-minute intervals, at Visit 1 or at Visit 2.
6. MI, unstable angina, coronary revascularization (percutaneous coronary intervention (PCI) or coronary artery bypass grafting (CABG)), ablation of atrial

flutter/fibrillation, valve repair/replacement within 12 weeks prior to enrolment. Before enrolment, these patients must have their qualifying echocardiography and/or cardiac MRI examination at least 12 weeks after the event.

7. Planned coronary revascularization, ablation of atrial flutter/fibrillation and valve repair/replacement.
8. Stroke or transient ischemic attack (TIA) within 12 weeks prior to enrolment.
9. Probable alternative or concomit
10. Body mass index >50 kg/m2.
